# SAM2-Dehaze: Fusing High-Quality Semantic Priors with Convolutions for Single-Image Dehazing

**DOI:** 10.3390/s25227097

**Published:** 2025-11-20

**Authors:** Sen Li, Jianchao Wang, Zhanqiang Huo

**Affiliations:** 1 School of Software, Henan Polytechnic University, Jiaozuo 454000, China; 212309010008@home.hpu.edu.cn; 2 China National Software & Service Company Limited, Beijing 102299, China; wangjc@css.com.cn

**Keywords:** image dehazing, SAM2, semantic priors, feature fusion

## Abstract

Single-image dehazing suffers from severe information loss and the under-constraint problem. The lack of high-quality robust priors leads to limited generalization ability of existing dehazing methods in real-world scenarios. To tackle this challenge, we propose a simple but effective single-image dehazing network by fusing high-quality semantic priors extracted from Segment Anything Model 2 (SAM2) with different types of advanced convolutions, abbreviated SAM2-Dehaze, which follows the U-Net architecture and consists of five stages. Specifically, we first employ the superior semantic perception and cross-domain generalization capabilities of SAM2 to generate accurate structural semantic masks. Then, a dual-branch Semantic Prior Fusion Block is designed to enable deep collaboration between the structural semantic masks and hazy image features at each stage of the U-Net. Furthermore, to avoid the drawbacks of feature redundancy and neglect of high-frequency information in traditional convolution, we have designed a novel parallel detail-enhanced and compression convolution that combines the advantages of standard convolution, difference convolution, and reconstruction convolution to replace the traditional convolution at each stage of the U-Net. Finally, a Semantic Alignment Block is incorporated into the post-processing phase to ensure semantic consistency and visual naturalness in the final dehazed result. Extensive quantitative and qualitative experiments demonstrate that SAM2-Dehaze outperforms existing dehazing methods on several synthetic and real-world foggy-image benchmarks, and exhibits excellent generalization ability.

## 1. Introduction

Single-image dehazing plays an important role in fields such as autonomous driving, intelligent surveillance, and remote sensing, serving as a crucial link between low-level image restoration and high-level visual understanding. Over the past decade, researchers have explored a wide range of approaches. Khan et al. [1] conducted a systematic review of traditional and deep learning-based dehazing methods and summarized the main challenges in this field. Prior-based physical models [2,3,4] estimate the parameters of the atmospheric scattering model based on statistical assumptions. While these methods can enhance image quality to some degree, their strong reliance on simplified physical priors limits their ability to handle complex and diverse real-world conditions. With the rise in convolutional neural networks (CNNs) [5] and transformers [6], data-driven methods [7,8,9] have gradually become the dominant trend. By directly learning degradation patterns directly from large-scale datasets, these methods have achieved remarkable progress and consistently outperform traditional techniques. However, since most deep learning models are trained on synthetic datasets, their performance often drops significantly when applied to real-world hazy images due to the domain gap, resulting in limited generalization ability.

**Figure 1:**
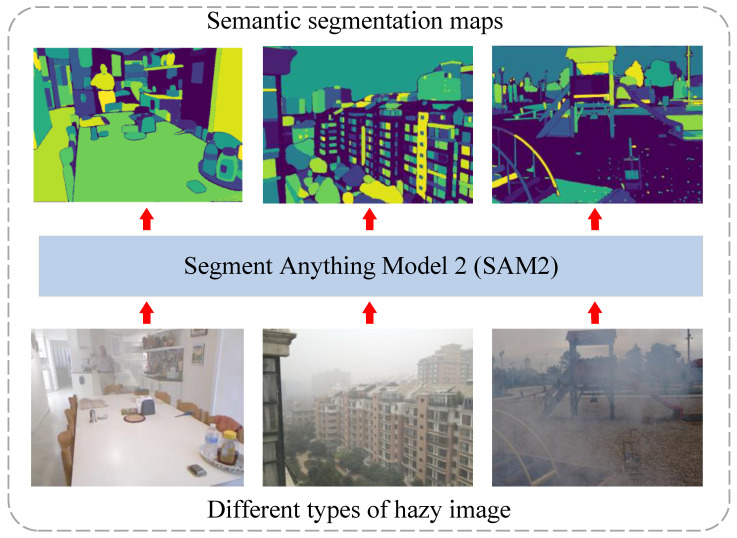
Illustration of SAM’s robustness on different types of hazy images. The figure demonstrates that SAM can accurately segment objects even when the input is a low-quality hazy image. This observation motivates us to leverage the semantic priors extracted from SAM, a large-scale foundation model, to enhance image restoration performance.

In recent years, the advancement of deep learning and large-scale pre-trained models have provided new directions for image dehazing. Among them, prior-guided approaches leveraging powerful vision foundation models have shown great potential. The Segment Anything Model (SAM) [10] and its successor SAM2 [11] demonstrate outstanding cross-domain generalization and rich semantic representations (see Figure 1), even under hazy conditions, providing reliable semantic priors for dehazing. Motivated by these capabilities, we propose SAM2-Dehaze, a semantic-aware dehazing framework that effectively integrates SAM2-derived priors to enhance detail recovery and visual consistency.

The main contributions of this work can be summarized as follows:

- We design a Semantic Prior Fusion Block (SPFB), which introduces SAM2-derived semantic information at multiple stages of the U-Net backbone. This semantic fusion mechanism guides the model to highlight structural features in key regions, enhancing its perception and restoration of edges and textures.
- We design a parallel detail-enhanced and compression convolution (PDCC), which combines standard, difference, and reconstruction convolutions to enable collaborative multi-level feature modeling. This module improves high-frequency detail representation while reducing redundancy.
- We design a Semantic Alignment Block (SAB) in the reconstruction phase, which performs fine-grained semantic alignment to restore colors, textures, and boundaries of key regions, thereby ensuring semantic consistency, visual naturalness, and structural integrity of the dehazed results.

The rest of this paper is organized as follows: Section 2 reviews related work on traditional, deep learning-based, and semantic-prior-guided image dehazing methods. Section 3 presents the architecture and implementation details of the proposed SAM2-Dehaze framework, including the Semantic Prior Fusion Block, the Parallel Detail-enhanced and Compression Convolution, and the Semantic Alignment Block. Section 4 reports the experimental settings, quantitative and qualitative results, and ablation studies to validate the effectiveness of each component. Section 5 concludes the paper by summarizing the main findings, discussing the limitations of the proposed method, and outlining directions for future research.

## 2. Related Work

### 2.1. Traditional Image Dehazing

In the early stages of image dehazing research, prior-based physical models dominated the field and became the mainstream approach. These methods typically rely on the atmospheric scattering model (ASM) and employ handcrafted empirical priors to model the statistical differences between hazy and haze-free images. By leveraging these priors, the transmission map and atmospheric light parameters are estimated to reconstruct a clear scene. For example, He et al. [12] proposed the Dark Channel Prior (DCP), which is based on the observation that in most local patches of outdoor haze-free images, at least a color channel exhibits very low intensity. This prior effectively facilitates the estimation of the transmission map and achieves impressive performance in dehazing tasks. Zhu et al. [13] introduced the Color Attenuation Prior, which analyzes the differences in brightness and saturation to establish a linear relationship with scene depth, enabling the inference of the transmission map. Berman et al. [14] proposed the Non-local Prior, which is based on the observation that pixels in the RGB color space tend to exhibit non-local clustering behavior. This structural property enables the effective separation of haze from scene content and provides reliable guidance for dehazing without explicit depth information.

Despite their early success and satisfactory performance in specific scenarios (e.g., buildings or ground regions), prior-based methods inherently suffer from the limitations of handcrafted assumptions. When dealing with complex and non-uniform haze distributions in natural scenes, handcrafted priors often fail to accurately capture the intricate relationships between haze and image content. Consequently, they tend to produce artifacts such as color distortion and halo effects, ultimately degrading the overall visual quality of dehazed images.

### 2.2. Deep Learning-Based Image Dehazing

With the rapid development of deep learning and the availability of large-scale synthetic dehazing datasets, learning-based image dehazing has rapidly become the mainstream research direction. Early deep-learning methods largely followed physics-guided paradigms, relying on the atmospheric scattering model (ASM) to estimate intermediate parameters such as transmission maps and atmospheric light for haze-free reconstruction. For instance, Shi et al. [15] proposed a zero-shot sand–dust image-restoration method based on the atmospheric scattering model, enabling unsupervised recovery of real sand–dust images without paired data and achieving superior visual quality. Ren et al. [16] proposed MSCNN to progressively refine transmission estimation; Li et al. [17] introduced AOD-Net to jointly predict transmission and atmospheric light in an end-to-end manner. To further improve estimation accuracy, Zhang et al. [18] designed DCPDN with dual-branch architecture, and Li et al. [19] enhanced model robustness under complex conditions through fuzzy region segmentation and haze-density decomposition.

Despite their effectiveness, physics-guided deep learning approaches are prone to cumulative errors in intermediate estimations, which may deteriorate final restoration quality. Consequently, recent research has shifted toward purely data-driven models that learn the dehazing process directly from data. For example, Liu et al. [20] developed GridDehazeNet based on attention mechanisms, Qin et al. [8] proposed FFA-Net with feature-level adaptive attention, and Wu et al. [21] introduced AECR-Net with contrastive regularization. Hong et al. [22] modeled prediction uncertainty via UDN, and Cheng et al. [23] presented DEA-Net for enhanced detail and structure preservation, while Wang et al. [24] proposed Dehaze-RetinetGAN by integrating Retinex theory with self-supervised learning. In addition, Son et al. [25] developed a Retinex-based multiscale training framework for sand–dust removal, achieving superior color fidelity and clarity under severe atmospheric conditions.

Although these methods significantly improve image clarity and visual quality, they still tend to overlook fine structural details. Many existing models primarily emphasize global color and brightness restoration, often resulting in blurred edges or semantic inconsistencies, which limits their performance in high-quality restoration tasks.

### 2.3. Semantic Priors for Image Dehazing

In recent years, several studies have incorporated semantic information to guide the dehazing process, aiming to enhance the modeling of image structure and content. For example, Zhang et al. [26] utilized a pre-trained DeepLabv3+ network to extract semantic features and integrated them into the dehazing network through an adaptive fusion module, thereby improving semantic awareness and regional discrimination. Cheng et al. [27] adopted the VGG16 network to extract semantic features and employed a global estimation module together with a color recovery module to transform semantic information into priors of object color and atmospheric light. Similarly, Song et al. [28] proposed a segmentation-guided framework that divides dehazing into prediction and restoration stages, significantly enhancing edge sharpness and texture reconstruction. Although these methods have achieved remarkable success in introducing structural priors and enhancing scene understanding, they generally rely on task-specific semantic segmentation models (e.g., DeepLabv3+ and VGG16) and require pre-training or fine-tuning on specific datasets, which to some extent limits their generalization ability.

The emergence of the Segment Anything Model (SAM) has changed this paradigm. As a large-scale pre-trained universal segmentation model, SAM shows strong structural perception and cross-domain generalization capabilities. It can produce high-quality masks without requiring extra supervision or task-specific training, which greatly expands the use of semantic priors in low-level vision tasks. For example, Zhang et al. [29] proposed an image restoration framework that uses semantic priors extracted from SAM to improve the model’s ability to capture both structural and semantic information, all without increasing inference costs. Li et al. [30] introduced the SAM-Deblur framework, the first to apply SAM to image deblurring. By using plug-and-play Mask Average Pooling (MAP) modules and a mask dropout strategy, they effectively added structural priors and improved generalization under non-uniform blur. Liu et al. [31] proposed SeBIR, a general framework for burst image restoration guided by SAM’s semantic priors. It uses a joint explicit–implicit alignment strategy and a semantic-guided fusion module, leading to significant improvements in alignment accuracy and multi-frame information integration.

Although SAM-based methods have shown great potential, most still utilize shallow semantic concatenation or simple fusion strategies, which fail to fully exploit SAM’s semantic priors in deep feature representation. This limitation becomes particularly evident in challenging scenes involving structural misalignment or regional degradation. To address these issues, we propose a structure-aware enhanced dehazing framework, SAM2-Dehaze, which fully leverages the rich semantic priors of SAM2. By designing three key modules, our method integrates semantic guidance throughout the network, achieving superior semantic consistency, detail recovery, and structural preservation.

## 3. Proposed Model

### 3.1. Overview of SAM2-Dehaze Model

**Figure 2:**
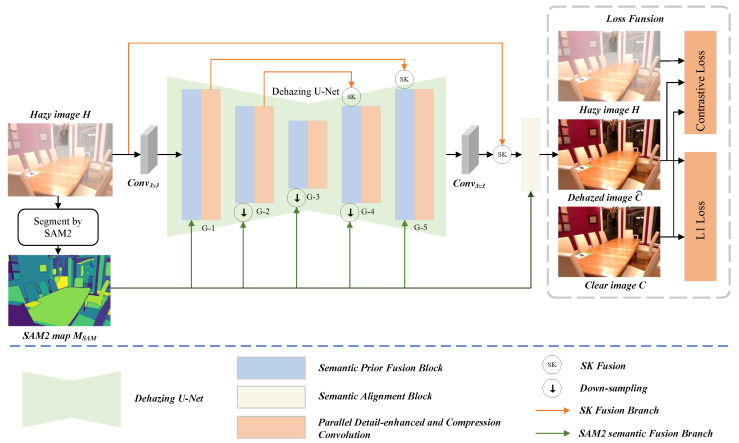
The architecture of SAM2-Dehaze. The input hazy image H is processed by SAM2 to generate a semantic map $M_{SAM}$. The backbone U-Net consists of five stages (G1–G5), each integrating Parallel Detail-enhanced and Compression Convolution (PDCC) and Semantic Prior Fusion Block (SPFB), and finally outputs a clear image through the Structure-Aware Block (SAB). During feature fusion at the G2, G3, and G4 stages, we down-sampling the $M_{SAM}$ using bilinear interpolation to ensure its size matches that of the fused features. The training uses a combination of L1 and contrastive losses.

We propose a semantic-guided image restoration framework named SAM2-Dehaze, which leverages semantic information to enhance the dehazing process. The overall architecture of the proposed model is illustrated in Figure 2. We first utilize a pre-trained SAM2 to generate semantic prior information, which is effectively injected into multiple stages (G1–G5) via the SPFB, thereby strengthening the network’s perception and understanding of semantic regions. Specifically, before applying SPFB at the G2, G3, and G4 stages, the semantic features are down-sampled using bilinear interpolation to align their spatial dimensions with the fused feature maps. However, considering that standard convolution has limited capability in capturing features around object boundaries and fine details, we introduce the Parallel Detail-enhanced and Compression Convolution module to enhance feature representation across semantic regions, compensating for the limitations of conventional convolution in edge and detail perception. To further improve the semantic consistency and structural fidelity of the dehazing results, we design a SAB. By modeling high-level relationships between the initially-restored image and the semantic priors, SAB performs structural-level semantic alignment, ensuring that the final dehazed image exhibits clearer edges, richer details, and stronger semantic coherence.

### 3.2. Semantic Prior Fusion Block

Due to its training on massive datasets and rich parameterization, SAM2 demonstrates exceptional segmentation capability across diverse scenarios and exhibits strong robustness to various types of image degradation. However, in image restoration tasks, directly using degraded images as input may hinder the model’s ability to accurately capture the true structural details of target regions. To address this, we leverage the semantic segmentation maps produced by SAM2 as prior information, providing rich and informative semantic cues to guide existing dehazing networks and thereby enhance overall restoration performance.

**Figure 3:**
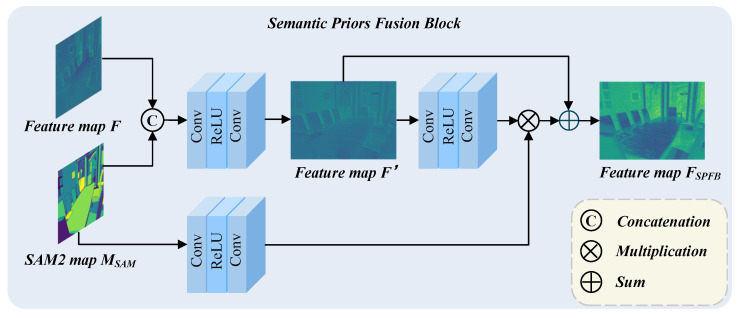
Architecture of the Semantic Prior Fusion Block (SPFB). The SPFB unit takes the semantic map $M_{SAM}$ and feature map *F* as input. After concatenation, they are processed to generate an intermediate feature $F^{\prime }$, which is then fused with guidance features from $M_{SAM}$ via element-wise multiplication. The output $F_{SPFB}$ is passed to subsequent network modules to enhance semantic representation and restoration quality.

First, we utilize the semantic map $M_{SAM}$ extracted by SAM2 as an explicit prior and fuse it with the feature map *F* of the low-quality image to enhance the feature representation capability of the restoration model. Specifically, as illustrated in Figure 3, the image feature *F* and the semantic prior $M_{SAM}$ are concatenated along the channel dimension and processed by a convolutional function g(·), which consists of two convolution layers and a ReLU activation, to generate the initial fused feature $F^{\prime }$:

(1) $$F^{\prime }=g\left(Concat\left(F,M_{SAM}\right)\right)$$

where Concat denotes the concatenation operation along the channel dimension. Next, we introduce a feature interaction mechanism to further enhance the integration of semantic information during restoration. Two parallel feature extraction branches are employed, each consisting of two convolutional layers and a ReLU activation, forming the convolutional function g(·). One branch extracts updated image features $F^{\prime }$, while the other refines semantic prior representations from $M_{SAM}$:

(2) $$F^{\prime \prime }=g\left(F^{\prime }\right)=g\left(g\left(Concat\left(F,M_{SAM}\right)\right)\right)$$

(3) $$P=g(M_{SAM})$$

where *P* represents the semantic feature extracted from the SAM segmentation map. To establish explicit interaction between image and semantic features, we apply element-wise multiplication between the outputs of the two branches, along with skip connections to preserve essential structural information. This adaptive design strengthens the network’s ability to focus on critical semantic regions and improves overall restoration consistency.

(4) $$F_{SPFB}=F^{\prime \prime }⊗P⊕F^{\prime }$$

where ⊗ denotes element-wise multiplication and ⊕ denotes element-wise addition.

### 3.3. Parallel Detail-Enhanced and Compression Convolution

Haze in natural scenes introduces variations in illumination and color, typically manifested in the loss of low-frequency components. Meanwhile, natural scenes covered by haze also tend to lose high-frequency details such as edges and contours. Traditional convolutional operations primarily focus on capturing low-frequency information while neglecting high-frequency restoration, which often becomes a performance bottleneck in dehazing performance. Moreover, standard convolutions easily introduce spatial redundancy, thereby reducing both efficiency and effectiveness. To overcome these limitations, we propose a parallel detail-enhanced and compression convolution module.

**Figure 4:**
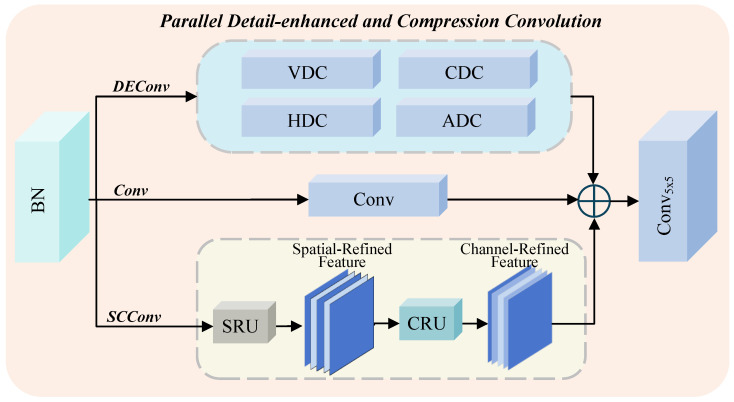
Architecture of the Parallel Detail-enhanced and Compression Convolution (PDCC). The module consists of a DEConv branch (VDC, HDC, CDC, ADC), a Conv branch, and an SCConv branch (SRU, CRU), which are responsible for detail extraction, basic feature learning, and spatial–channel refinement, respectively. The outputs are fused and further processed by a 5 × 5 convolution to generate the enhanced feature representation.

The PDCC module consists of three parallel branches: the Detail Enhancement Convolution (DEConv) [23] branch, the Convolution (Conv) Branch, and the Spatial-Channel Construction Convolution (SCConv) [32] branch, as illustrated in Figure 4. We first apply Batch Normalization to the input feature $F_{SPFB}$ to obtain the normalized input $F_{in}=BatchNorm\left(F_{SPFB}\right)$. The normalized features are then fed into the three branches for independent processing. Specifically, the Conv branch is responsible for basic feature extration and the preservation of low-frequency information. The DEConv branch focuses on multi-directional high-frequency detail extraction and incorporates four types of difference convolutions: Center Difference Convolution (CDC), Angle Difference Convolution (ADC), Horizontal Difference Convolution (HDC), and Vertical Difference Convolution (VDC). These convolutions capture high-frequency details at multiple scales and orientations. However, directly applying them significantly increases the number of parameters and computational cost. To address this issue, following [23], we combine these convolution kernels at corresponding positions to form an equivalent kernel, thereby maintaining rich feature extraction capability while reducing computational complexity. The specific operations are as follows:

(5) $$DEConv\left(F_{in}\right)=\sum_{i=1}^{4}f_{K_{i}}\left(F_{in}\right)=f_{K_{cvt}}\left(F_{in}\right)$$

where $K_{i}$ represents the kernels used in the *i*th convolution operations, $F_{in}$ denotes the input feature map, $f_{k}\left(\cdot \right)$ denotes the convolution operation with a kernel of size *k*, and $K_{cvt}$ denotes the equivalent kernel obtained by combining the four kernels. The SCConv is designed to suppress spatial redundancy and enhance feature representation capability. It comprises two sequential components: a Spatial Reconstruction Unit (SRU) and a Channel Reconstruction Unit (CRU). The SRU reduces spatial redundancy using a “separation-and-reconstruction” strategy, while the CRU employs a “split-transform-fuse” mechanism to minimize channel redundancy. The specific operations are as follows:

(6) $$X_{w}=SRU\left(F_{in}\right)$$

(7) $$SCConv\left(F_{in}\right)=CRU\left(SRU\left(F_{in}\right)\right)=CRU\left(X_{w}\right)$$

where $X_{w}$ denotes the feature map after spatial reconstruction. Finally, the outputs from the Conv, DEConv, and SCConv branches are aggregated and refined using a 5 × 5 convolution layer to produce the final output feature $F_{out}$:

(8) $$F_{out}=Conv_{5\times 5}\left(\mathrm{Conv}\left(F_{in}\right)+DEConv\left(F_{in}\right)+SCConv\left(F_{in}\right)\right)$$

This parallel architecture enables the PDCC module to effectively recover both low- and high-frequency information, enhance structural detail perception, and suppress redundancy, thereby improving the overall performance and efficiency of the dehazing network.

### 3.4. Semantic Alignment Block

Semantic information plays a crucial role in restoring degraded images, particularly in reconstructing color, contrast, and texture consistency. Objects belonging to the same semantic category often share similar structural and visual characteristics. These intra-class semantic correlations can effectively constrain the solution space of image restoration, facilitating the preservation of both global structures and fine-grained details. To leverage this property, we design a Semantic Alignment Block in the post-processing phase of the dehazing network, where high-level semantics act as structural priors for further refinement. The SAB extracts key semantic features from the semantic segmentation map generated by SAM2 and fuses them with the structural representations of the coarsely dehazed image. This deep semantic–structural integration improves the network’s ability to recognize semantic regions and maintain structural integrity, thereby enhancing the clarity, naturalness, and semantic coherence of the final restored image.

**Figure 5:**
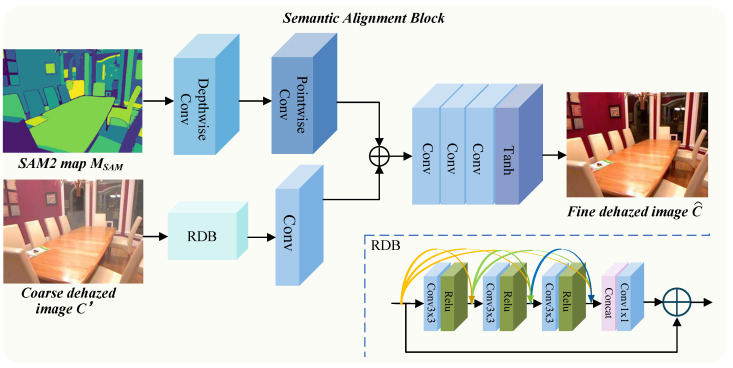
Detailed structure of the Semantic Alignment Block. The generated close dehazed image is fused with the semantic-guided features to produce a finer dehazed result. Benefiting from high-level semantic information, the refined dehazed image can better preserve the structure, color, and details of objects.

Specifically, as illustrated in Figure 5, depth-wise separable convolutions are first applied to the semantic segmentation map $M_{SAM}$ obtained from SAM2 to extract semantic-guided features. Subsequently, a Residual Dense Block (RDB) [33] is applied to the coarsely dehazed image $C^{\prime }$ to obtain refined image features. The RDB serves an efficient residual dense module that enhances the representation of structural and fine details through residual learning and dense connections. Next, the semantic-guided features and the image features from the coarse dehazing stage are fused to enable semantic–structural alignment. To further refine the fused representation, multiple standard convolutional layers followed by a hyperbolic tangent (Tanh) activation function are employed to generate the final dehazed image $\hat{C}$. With the guidance of high-level semantic priors provided by SAM2, the proposed framework can effectively recover semantically consistent, clearer, and more natural haze-free images.

### 3.5. Train Loss

During the training process, we adopt the L1 loss and contrastive loss to optimize the quality of the dehazed images. The L1 loss is used to directly constrain the pixel-level difference between the dehazed image and the ground-truth haze-free image, while the contrastive loss enforces the similarity of deep feature representations to improve structural fidelity and perceptual quality. Specifically, given a hazy image H and its corresponding clear image *C*, we denote the predicted dehazed image from our SAM2-Dehaze as $\hat{C}$. The contrastive loss optimization objective can be formulated as

(9) $$L_{c}=\sum_{i=0}^{n}w_{i}\cdot \frac{\|R_{i}\left(C\right),R_{i}\left(\hat{C}\right){\|}_{1}}{\|R_{i}\left(H\right),R_{i}\left(\hat{C}\right){\|}_{1}}$$

where $R_{i}\left(\cdot \right)\;\left(i=1,2,\ldots ,n\right)$ denotes the features extracted from the *i* layer of a fixed pre-trained model and $w_{i}$ represents the corresponding weight coefficient for that layer. In our study, we extract features from layers 11, 35, 143, and 152 in the ResNet-152 [5] and set the weights $w_{i}\left(i=1,\ldots ,4\right)$ to $\frac{1}{16},\frac{1}{8},\frac{1}{4}$ and 1, respectively. In fact, previous research [34] has demonstrated that, in image restoration tasks, compared with the L2 norm, the L1 norm yields better performance. The L1 loss is defined as

(10) $$L_{1}=\|C-\hat{C}\|=\frac{1}{N}\sum_{j=1}^{N}|C_{j}-\hat{C_{j}}|$$

where N denotes the total number of pixels in the image and $\hat{C_{j}}$ and $C_{j}$ represent the predicted dehazed result and the *j* pixel values of the ground-truth haze-free image, respectively. By combining the L1 loss and contrastive loss, our final loss function is defined as

(11) $$L=L_{1}+\lambda L_{c}$$

where λ is the hyperparameter used to balance the two loss terms and is set to 0.1 in our experiments.

## 4. Experiments and Results

### 4.1. Datasets and Evaluation Metrics

Datasets: We conduct a comprehensive evaluation of the proposed method on both synthetic and real-world image datasets. For synthetic data, we employ the Realistic Single Image Dehazing (RESIDE) [35] dataset, which is a widely used benchmark comprising multiple subsets, including the Indoor Training Set (ITS), Outdoor Training Set (OTS), Synthetic Objective Testing Set (SOTS), and Hybrid Subjective Testing Set (HSTS). In the synthetic experiments, our model is trained on ITS and OTS, and evaluated on SOTS-indoor and SOTS-outdoor, respectively. For real-world scenarios, we adopt the Dense-Haze [36], NH-Haze [37], and RTTS datasets to assess the model’s performance on natural hazy scenes. The detailed experimental configurations are summarized in Table 1.

Evaluation Metrics: To quantitatively assess dehazing performance, we employ seven widely used image quality evaluation metrics, which are categorized into full-reference and no-reference types. The full-reference metrics include the Peak Signal-to-Noise Ratio (PSNR) [38,39], Structural Similarity Index (SSIM) [40,41], and CIEDE2000 color difference [42]. The no-reference metrics consist of Fog Aware Density Evaluation (FADE) [43], Natural Image Quality Evaluator (NIQE) [44], Perception-based Image Quality Evaluator (PIQE) [45], and Blind/Referenceless Image Spatial Quality Evaluator (BRISQUE) [46]. These metrics are widely adopted in computer vision to measure both visual quality and perceptual consistency of images. In addition to image quality evaluation, we also assess network efficiency using the number of parameters (Param, in millions), computational complexity (MACs, in billions of multiply–accumulate operations), and inference latency (Latency, in milliseconds). To ensure fairness, all experiments are conducted on images with a resolution of 250 × 250.

### 4.2. Implementation Details

We use the SAM2 pre-trained model for segmentation. Compared with the SAM segmentation model, SAM2 demonstrates significant improvements in segmentation accuracy, speed, interaction efficiency, and applicability across various scenarios. In particular, it shows stronger information processing capabilities and generalization in zero-shot segmentation tasks. All experiments are conducted on an NVIDIA RTX A6000 GPU (48 GB VRAM), and the model is implemented based on the Pytorch 2.4.1 framework. During training, we optimize the network using the AdamW optimizer, with decay parameters set to $\beta_{1}=0.9$ and $\beta_{2}=0.999$. The initial learning rate is set to $2\times 10^{-4}$, and it is gradually decreased to $2\times 10^{-6}$ using a cosine annealing strategy to ensure training stability. During training, input images are randomly cropped into 256 × 256 patches. We design three variants of SAM2-Dehaze, named S (Small), B (Basic), and L (Large). Table 2 presents detailed configurations of each variant, with two key columns indicating the number of G-blocks in the network and their corresponding embedding dimensions.

### 4.3. Comparison with State of the Arts

In this section, we compare our SAM2-Dehaze with eleven dehazing methods, including DCP [12], MSCNN [16], AOD-Net [17], GridDehazeNet [20], FFA-Net [8], AECR-Net [21], Dehamer [47], MIT-Net [48], RIDCP [49], C2PNet [50], and DEA-Net [23]. For synthetic datasets (SOTS-indoor and SOTS-outdoor), all methods are trained and evaluated under identical experimental conditions. For real-world datasets, we compare seven methods (DCP, AOD-Net, FFA-Net, Dehamer, MIT-Net, RIDCP, MixDehazeNet [51]) on the Dense-Haze and NH-Haze datasets. Additionally, for the RTTS dataset, we benchmark our method against seven advanced models, including GridDehazeNet, FFA-Net, Dehamer, C2PNet, MIT-Net, DEA-Net, and IPC-Dehaze [52]. On synthetic datasets, we report results for three variants of SAM2-Dehaze (-S, -B, -L), while on the real-world datasets, only the SAM2-Dehaze-L variant is evaluated. To ensure a fair comparison, we use either the officially released code or the publicly reported results of existing methods. If these are unavailable, we retrain the models under the same dataset and parameter settings as our approach.

Results on Synthetic Datasets: Table 3 presents the quantitative evaluation results of our SAM2-Dehaze and other state-of-the-art methods on the SOTS dataset. As shown, the SAM2-Dehaze-L achieves the best performance on the SOTS-indoor dataset, reaching a PSNR of 42.83 dB and an SSIM of 0.997, outperforming all other methods. Even the lightweight SAM2-Dehaze-S variant ranks second, with a PSNR of 41.41 dB and an SSIM of 0.996. On the SOTS-outdoor dataset, our method does not achieve the best result but still ranks in the upper-middle tier, with the SAM2-Dehaze-L variant achieving a PSNR of 36.22 dB and SSIM of 0.989. In addition, as shown Table 4, we report Params, MACs, and Latency as the main metrics for computational efficiency. Compared with recent advanced methods, SAM2-Dehaze achieves a balanced trade-off between accuracy and efficiency. Given the performance improvements, the slight increase in computational cost is acceptable. All efficiency metrics, including Params, MACs, and Latency, are computed using 256 × 256 RGB input images, ensuring fair and consistent evaluation.

**Figure 6:**
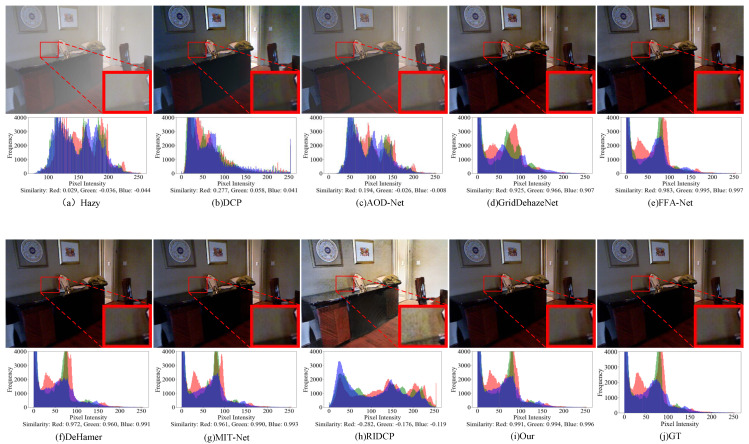
Visual and histogram results of SOTS−indoor dataset by different methods. Zoom in for best view.

**Figure 7:**
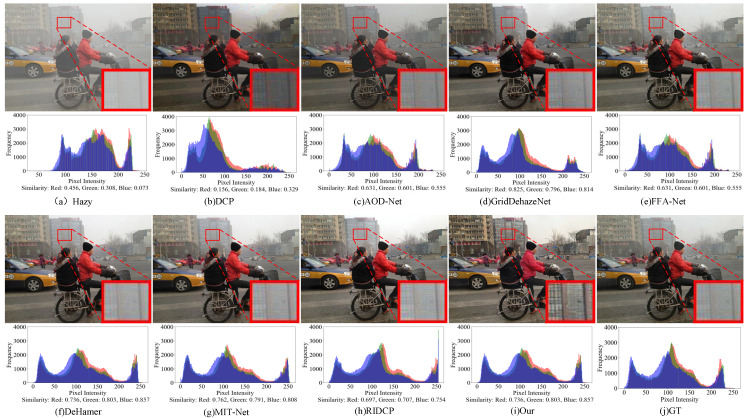
Visual and histogram results of SOTS−outdoor dataset by different methods. Zoom in for best view.

Figure 6 and Figure 7 present the visual comparisons between our proposed method and several state-of-the-art dehazing algorithms on the SOTS-indoor and SOTS-outdoor datasets. To provide a more comprehensive evaluation, we additionally analyze the RGB histogram distributions of the restored images, enabling quantitative assessment of color consistency alongside subjective visual inspection. On the SOTS-indoor dataset, traditional methods such as DCP and RIDCP perform poorly, exhibiting noticeable color distortions and residual artifacts in the reconstructed images. AOD-Net also struggles to remove haze effectively. Although GridDehazeNet, FFA-Net, Dehamer, and MIT-Net produce visually appealing results, our method achieves RGB distributions that align more closely with the ground-truth clear images, demonstrating clear advantages in both color restoration and detail preservation. For the SOTS-outdoor dataset, DCP often introduces sky region artifacts, while AOD-Net retains partial structural information yet leaves visible haze residuals. It is important to note that SOTS-outdoor is a synthetic dataset generated based on the atmospheric scattering model, and some nominally “haze-free” images may still contain subtle haze traces, which can introduce training noise and bias the evaluation. While methods such as GridDehazeNet, FFA-Net, Dehamer, MIT-Net, and RIDCP perform competitively on synthetic datasets, they exhibit limitations in handling residual real-world haze embedded in the scenes. In contrast, our proposed SAM2-Dehaze not only removes synthetic haze but also demonstrates stronger robustness and adaptability when dealing with complex or residual real-world haze, thereby achieving superior performance in both visual fidelity and detail preservation.

Results on Real-World Datasets: Table 5 presents the quantitative comparison between our SAM2-Dehaze-L and several state-of-the-art methods on the Dense-Haze and NH-Haze datasets. As shown, our method achieves superior and stable performance across both datasets. On the Dense-Haze dataset, our method attains the best results, achieving a PSNR of 20.61 dB, SSIM of 0.725, and CIEDE2000 of 8.5909. Compared with the baseline MixDehazeNet-L, our method approach improves PSNR by 4.71 dB, SSIM by 0.146, and CIEDE2000 by 28.99%. On the NH-Haze dataset, our method also achieves the best results, with a PSNR of 22.02 dB, SSIM of 0.831, and CIEDE2000 of 8.5108. Although the improvement margin is smaller: 1.01 dB in PSNR, 0.004 in SSIM, and 10.53% in CIEDE2000. This smaller gain can be attributed to the higher haze concentration and stronger degradation in the Dense-Haze dataset, which presents more significant challenges for conventional feature extraction. In contrast, our method effectively leverages the semantic priors provided by SAM2, substantially enhancing the network’s structural perception and semantic representation under dense-haze conditions. Consequently, SAM2-Dehaze-L achieves more robust and reliable restoration results, demonstrating significant improvements in visual quality and quantitative performance.

**Figure 8:**
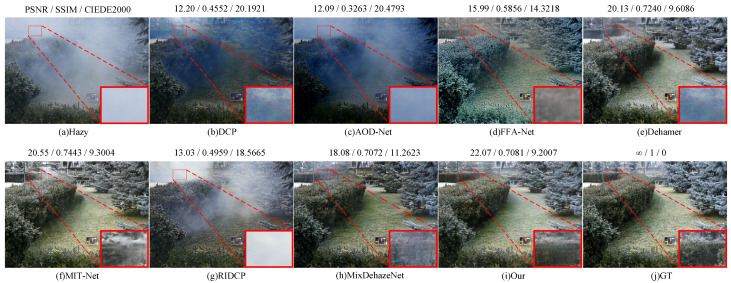
Visual results of NH datasets by different methods. Zoom in for best view.

**Figure 9:**
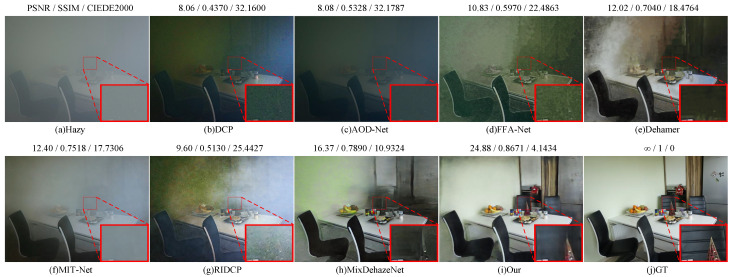
Visual results of Dense datasets by different methods. Zoom in for best view.

Figure 8 and Figure 9 illustrate the qualitative comparison results of our method on the Dense-Haze and NH-Haze datasets. Traditional methods such as DCP and RIDCP exhibit noticeable color distortions and residual haze on both datasets. Lightweight deep models like AOD-Net show limited dehazing capability, often failing to recover clear structures in dense-haze regions. While FFA-Net and MIT-Net achieve better visual quality, they struggle with detail preservation, resulting in blurry local textures. Dehamer enhances overall brightness but introduces artifacts and unnatural color tones, whereas MixDehazeNet achieves more balanced performance yet still fails to handle regions with heavy haze accumulation. In contrast, our proposed SAM2-Dehaze achieves superior dehazing performance on both datasets. It effectively restores realistic color, fine textures, and structural consistency, while suppressing noise and residual artifacts. The resulting images appear visually clearer, more natural, and highly consistent with the ground-truth references, confirming the robustness and generalization of our approach under challenging real-world haze conditions.

Results on RTTS datasets: Table 6 presents the quantitative comparison between our method and several state-of-the-art dehazing approaches on the RTTS dataset, evaluated using four no-reference image quality metrics: FADE, NIQE, PIQE, and BRISQUE. As shown in the table, although IPC-Dehaze achieves the best overall performance, our method outperforms most existing methods across all metrics.

**Figure 10:**
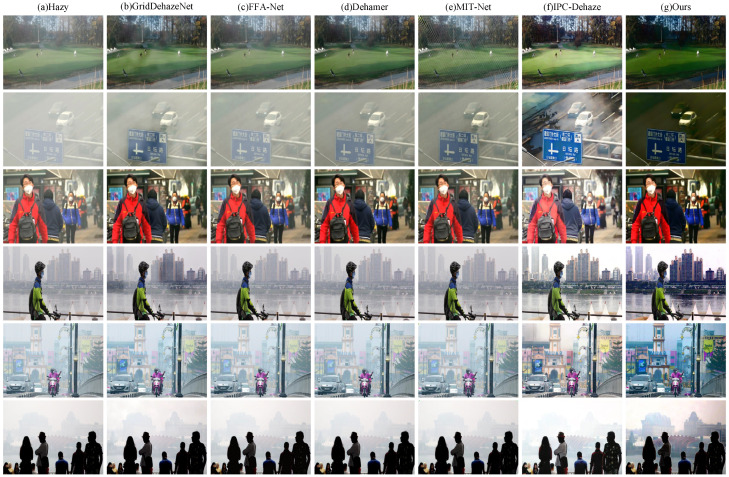
Visual comparison on RTTS. Zoom in for best view.

To further assess the qualitative performance, Figure 10 provides a visual comparison on the RTTS dataset. Overall, FFA-Net, Dehamer, GridDehazeNet, and MIT-Net exhibit limited dehazing capability under complex real-world conditions, with noticeable residual haze persisting in the reconstructed images. ICP-Dehaze demonstrates a certain level of effectiveness in some samples but still falls short in terms of structural detail recovery and color fidelity. In contrast, our method consistently delivers superior visual quality across all test images. The restored results are significantly cleaner and more visually pleasing, with natural color reproduction and well-preserved details, without introducing noticeable artifacts or over-enhancement effects.

### 4.4. Ablation Study

Impact of Different Components in the Network. To further validate the effectiveness of each proposed component, we conduct ablation studies to analyze the contributions of the key modules, including the SPFB, the PDCC, and the SAB. We take MixDehazeNet-S as the baseline network and construct four variants based on it as follows:

(1) Base + SPFB → V1
(2) Base + HEConv → V2
(3) Base + SPFB + HEConv → V3
(4) Base + SPFB + HEConv + SAB → V4

**Figure 11:**
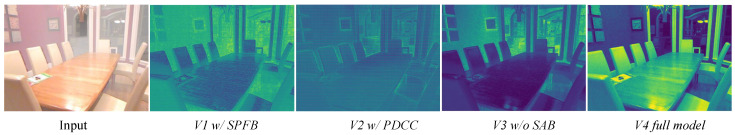
Visual comparison of intermediate features in ablation models.

All models are trained using the same training strategy, and the “S” variant is evaluated on the ITS-indoor test set. The experimental results are shown in Table 7 and Figure 11. As illustrated in Table 7, each proposed module contributes notably to the overall improvement in dehazing performance. Specifically, the SPFB module increases PSNR by 1.77 dB compared with the baseline. Similarly, the PDCC module introduces additional gains by improving fine-grained edge and structure restoration. Overall, each component plays a complementary role in enhancing dehazing quality, verifying the effectiveness of our architectural design. To further illustrate the contribution of each module, we visualize their respective feature maps. As shown: V1⁠w/⁠SPFB enhances some edge features but still suffers from coarse outputs and insufficient detail recovery; V2⁠w/⁠PDCC improves edge and structure clarity to some extent but lacks fine-grained textures; V3⁠w/o⁠SAB yields moderate global improvement but fails to preserve table textures and background structures, resulting in blurry features. In contrast, the V4fullmodel, which integrates SPFB, PDCC, and SAB, produces much clearer features with better spatial sharpness and detail recovery, including more precise object boundaries. These visualizations further confirm the effectiveness of each proposed component.

Ablation Study on SPFB Module. To thoroughly validate the effectiveness of the proposed SPFB module, we conducted a series of detailed ablation experiments from two perspectives: external fusion strategies and internal structural variations in the SPFB module.

(1) External fusion strategies

- SPFB-N1: The SPFB module is removed, and feature fusion is performed via simple element-wise addition.
- SPFB-N2: The input RGB image is extended to four channels by appending the semantic segmentation mask as an additional channel before feeding it into the dehazing network.

(2) Internal structural variations

- SPFB-F1: The fusion with input feature *F* is removed, and only the intermediate semantic attention map $M_{\mathrm{SAM}}$ is used within function g(·).
- SPFB-F2: The feature extraction branch responsible for obtaining semantic priors from $M_{\mathrm{SAM}}$ is removed.

As shown in the results Table 8, none of these alternatives, whether in terms of fusion strategies or SPFB structural variants, can match the performance of our original SPFB design. This clearly demonstrates the superior capability of our SPFB module in both structural fusion and semantic guidance. Moreover, we further analyzed the impact of inserting the SPFB module at different positions in the network. As illustrated in Table 8, increasing the number of inserted SPFB modules consistently improves the network performance. This progressive enhancement trend further confirms the effectiveness of the SPFB module, especially in boosting the modeling capacity of multi-level features through structural and semantic reinforcement.

**Figure 12:**
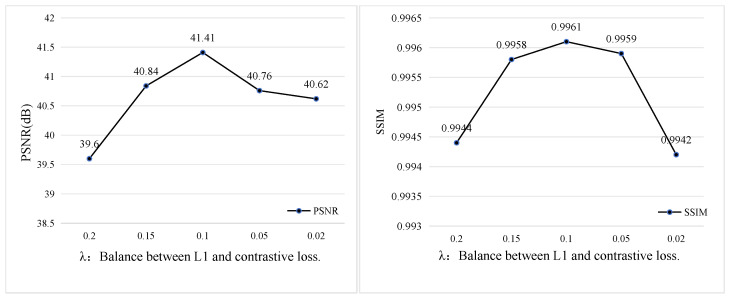
Effect of the λ parameter on dehazing performance. The plots illustrate the impact of different λ values—used to balance the L1 loss and contrastive loss—on PSNR (**left**) and SSIM (**right**). The best performance is achieved when λ = 0.1, yielding the highest PSNR of 41.41 dB and the highest SSIM of 0.9961.

Impact of Loss Function Hyperparameters. To select the optimal hyperparameters, we conduct ablation experiments on the weight parameters of the contrastive loss and L1 loss. A penalty factor λ lambda is introduced before the contrastive loss to determine its optimal contribution. As shown in Figure 12, when λ=0.1, the model achieves the best performance in both PSNR and SSIM, indicating the most effective dehazing capability.

## 5. Conclusions

This paper addresses the limitations of traditional image dehazing methods in semantic understanding and detail restoration by proposing a novel dehazing framework, SAM2-Dehaze, which integrates semantic prior information from a large-scale pre-trained model. We incorporate the SAM2 model to fully exploit its powerful capabilities in semantic segmentation and structural perception, and design, three key modules: the Semantic Prior Fusion Block (SPFB), the Parallel Detail-enhanced and Compression Convolution (PDCC), and the Semantic Alignment Block (SAB). These modules work collaboratively to significantly enhance semantic consistency, detail preservation, and structural reconstruction during the dehazing process. Extensive experiments on multiple standard hazy-image datasets demonstrate that the proposed method outperforms existing state-of-the-art approaches in both quantitative metrics and subjective visual quality, showing strong generalization and practical potential.

**Figure 13:**
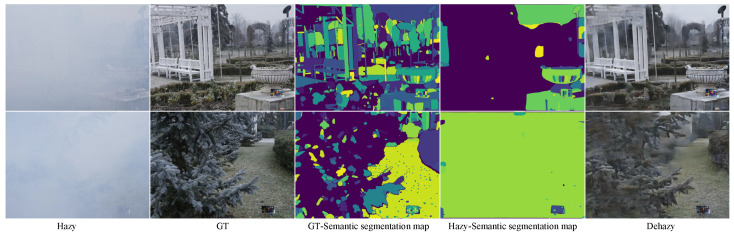
Semantic segmentation and dehazing results of SAM2-Dehaze. The model’s dependence on a pre-trained semantic segmentation network restricts its performance in extremely dense-haze and resource-limited scenarios.

Although SAM2-Dehaze exhibits excellent performance and a strong generalization ability, it still depends on a pre-trained semantic segmentation model, which may introduce additional computational overhead in resource-constrained environments. Future work will explore lightweight semantic embedding strategies and self-distillation mechanisms to reduce dependence on large-scale foundation models and further improve real-time performance. Moreover, when the haze in an image is excessively dense, even powerful large-scale models such as SAM2 struggle to achieve accurate segmentation. The lack of sufficient semantic information further limits the dehazing performance of the model (as shown in Figure 13), which remains one of the key challenges to be addressed in future research.

## Figures and Tables

**Table 1:** The details of the datasets used in our experiments. ITS–L represents a model of type L trained on the ITS dataset.

Datasets	Train (GT/Hazy)	Test (GT/Hazy)	Train Epochs	Pre-Trained Weights
RESIDE	ITS (1399/13,990)	SOTS-indoor (500/500)	500	–
	OTS (8970/313,950)	SOTS-outdoor (500/500)	50	–
		RTTS (4322)	50	–
Dense-Haze	Dense-Haze (45/45)	Dense-Haze (5/5)	5000	ITS–L
NH-Haze	NH-Haze (45/45)	NH-Haze (5/5)	5000	ITS–L

**Table 2:** Model Architecture Detailed.

Model Name	Num. of Blocks	Embedding Dims
SAM2-Dehaze-S	[2, 2, 4, 2, 2]	[24, 48, 96, 48, 24]
SAM2-Dehaze-B	[4, 4, 8, 4, 4]	[24, 48, 96, 48, 24]
SAM2-Dehaze-L	[8, 8, 16, 8, 8]	[24, 48, 96, 48, 24]

**Table 3:** Quantitative comparison on SOTS-indoor/outdoor. We report PSNR, SSIM and CIEDE2000. The ↑ indicates that a larger value is better, the ↓ indicates that a smaller value is better. The symbol “— ” indicates that the value is unavailable. Bold and underlined values represent the best and second-best results.

Method	SOTS-Indoor			SOTS-Outdoor		
	PSNR↑	SSIM↑	CIEDE2000↓	PSNR↑	SSIM↑	CIEDE2000↓
DCP (TPAMI’10)	16.61	0.855	6.7998	19.14	0.861	11.0133
MSCNN (ECCV’16)	19.84	0.833	7.6254	22.06	0.908	6.2546
AOD-Net (ICCV’17)	20.51	0.816	8.0860	24.14	0.920	8.9375
GridDehazeNet (ICCV’19)	32.16	0.984	1.7784	30.86	0.960	2.2747
FFA-Net (AAAI’20)	36.39	0.989	1.1645	33.38	0.984	2.4797
AECR-Net (CVPR’21)	37.17	0.990	1.1423	—	—	—
Dehamer (CVPR’22)	36.63	0.988	0.9881	35.18	0.986	**0.9676**
MIT-Net (MM’23)	40.23	0.992	0.9920	35.18	0.988	0.9800
RIDCP (CVPR’23)	18.36	0.757	9.9759	21.62	0.833	7.9011
C2PNet (CVPR’23)	42.46	0.995	0.6997	**36.68**	**0.990**	0.9762
DEA-Net (TIP’24)	41.21	0.992	0.7994	36.24	0.989	0.9771
SAM2-Dehaze-S	41.41	0.996	0.7766	35.62	0.982	1.1625
SAM2-Dehaze-B	41.56	0.996	0.7526	35.69	0.985	0.9956
SAM2-Dehaze-L	**42.83**	**0.997**	**0.6929**	36.22	0.989	0.9823

**Table 4:** Computational efficiency comparison. Bold and underlined values denote the best and second-best results, respectively.

Method	Overhead		
	Param. (M)	MACs (G)	Latency (ms)
GridDehazeNet (ICCV’19)	**0.96**	21.43	39.69
FFA-Net (AAAI’20)	4.45	287.53	164.94
AECR-Net (CVPR’21)	2.61	52.20	36.26
Dehamer (CVPR’22)	132.45	48.93	—
MIT-Net (MM’23)	2.73	**16.54**	**14.57**
C${⁠}^{2}$ PNet (CVPR’23)	7.17	460.95	173.86
DEA-Net (TIP’24)	3.65	34.04	16.12
SAM2-Dehaze-S	5.06	43.07	97.93
SAM2-Dehaze-B	9.78	71.36	242.21
SAM2-Dehaze-L	19.24	127.96	519.89

**Table 5:** Quantitative comparison on the Dense-Haze and NH-Haze datasets. The ↑ indicates that a larger value is better, the ↓ indicates that a smaller value is better. Bold and underlined values denote the best and second-best results, respectively.

Method	Dense-Haze			NH-Haze		
	PSNR↑	SSIM↑	CIEDE2000↓	PSNR↑	SSIM↑	CIEDE2000↓
DCP (TPAMI’10)	11.06	0.417	23.5067	13.28	0.482	18.0389
AOD-Net (ICCV’17)	12.82	0.468	24.0294	15.69	0.573	19.3886
FFA-Net (AAAI’20)	16.24	0.561	13.8080	16.29	0.562	13.1681
DeHamer (CVPR’22)	16.63	0.587	12.8506	20.66	0.686	9.1162
MIT-Net (MM’23)	16.97	0.623	12.5450	21.25	0.712	8.5118
RIDCP (CVPR’23)	8.09	0.438	32.2540	12.27	0.503	20.2104
MixDehazeNet-L (IJCNN’24)	15.90	0.579	12.0986	21.01	0.827	9.5122
SAM2-Dehaze-L	**20.61**	**0.725**	**8.5909**	**22.02**	**0.831**	**8.5108**

**Table 6:** Quantitative comparison on RTTS. The ↓ indicates that a smaller value is better. Bold and underlined values indicate the best and second-best results, respectively.

Method	FADE ↓	NIQE ↓	PIQE ↓	BRISQUE ↓
GridDehazeNet (ICCV’19)	1.72	4.85	23.85	29.73
FFA-Net (AAAI’20)	2.07	4.93	24.59	34.44
Dehamer (CVPR’22)	1.92	4.91	23.31	34.55
C2PNet (CVPR’23)	2.06	5.03	25.05	34.80
MIT-Net (MM’23)	1.97	4.92	23.25	34.37
DEA-Net (TIP’24)	1.90	4.92	24.95	31.99
IPC-Dehaze (CVPR’25)	**1.15**	**4.08**	**12.34**	**24.79**
SAM2-Dehaze (Ours)	1.71	4.75	23.23	30.09

**Table 7:** Ablation study on the RESIDE-indoor dataset. The ✓ indicates that the corresponding module is enabled, while w/o denotes that the module is not used. Bold values indicate the best results.

Variants	Baseline	V1	V2	V3	V4
MixDehazeNet-S	✓	✓	✓	✓	✓
SPFB	w/o	✓	w/o	✓	✓
HEConv	w/o	w/o	✓	✓	✓
SAB	w/o	w/o	w/o	w/o	✓
PSNR	39.47	41.24	40.26	41.37	**41.41**
SSIM	0.995	0.996	0.995	0.996	**0.996**

**Table 8:** Ablation study on different fusion methods (left) and SPFB insertion locations (right). Bold values indicate the best results.

Variants			Location		
Method	PSNR	SSIM	Block	PSNR	SSIM
SPFB-N1	38.91	0.994	G-1	40.16	0.995
SPFB-N2	40.26	0.995	G-2	40.66	0.995
SPFB-F1	41.14	0.996	G-3	40.89	0.995
SPFB-F2	41.24	0.996	G-4	41.31	0.996
SPFB	**41.41**	**0.996**	G-5	**41.41**	**0.996**

## Data Availability

The datasets used in this study are publicly available. RESIDE (including ITS/OTS/SOTS/RTTS and related subsets) can be accessed at the authors’ project site: RESIDE: https://sites.google.com/view/reside-dehaze-datasets (accessed on 12 January 2025). Dense-HAZE (NTIRE 2019) is available at https://data.vision.ee.ethz.ch/cvl/ntire19/dense-haze (accessed on 12 January 2025). NH-HAZE (NTIRE 2020) is available at https://data.vision.ee.ethz.ch/cvl/ntire20/nh-haze (accessed on 12 January 2025).

## Abbreviations

The following abbreviations are used in this manuscript:

SAM	Segment Anything Model
SPFB	Semantic Prior Fusion Block
PDCC	Parallel Detail-enhanced and Compression Convolution
SAB	Semantic Alignment Block
ASM	Atmospheric Scattering Model
DCP	Dark Channel Prior
U-net	U-shaped convolutional neural network
VGG16	Visual Geometry Group 16-layer network
ReLU	Rectified Linear Unit
DEConv	Detail Enhancement Convolution
CDC	Center Difference Convolution
ADC	Angle Difference Convolution
HDC	Horizontal Difference Convolution
VDC	Vertical Difference Convolution
SCConv	Spatial-Channel Construction Convolution
SRU	Spatial Reconstruction Unit
CRU	Channel Reconstruction Unit
RDB	Residual Dense Block
PSNR	Peak Signal-to-Noise Ratio
SSIM	Structural Similarity Index
FADE	Fog Aware Density Evaluation
NIQE	Natural Image Quality Evaluator
PIQE	Perception-based Image Quality Evaluator
BRISQUE	Blind/Referenceless Image Spatial Quality Evaluator

## References

[1] Khan H., Xiao B., Li W., Muhammad N. (2022). Recent Advancement in Haze Removal Approaches. Multimed. Syst..

[2] Ju M., Ding C., Ren W., Yang Y., Zhang D., Guo Y.J. (2021). IDE: Image Dehazing and Exposure Using an Enhanced Atmospheric Scattering Model. IEEE Trans. Image Process..

[3] Wang X., Chen X.A., Ren W., Han Z., Fan H., Tang Y., Liu L. (2024). Compensation Atmospheric Scattering Model and Two-Branch Network for Single Image Dehazing. IEEE Trans. Emerg. Top. Comput. Intell..

[4] Xin W., Xudong Z., Jun Z., Rui S. (2025). Image Dehazing Algorithm by Combining Light Field Multi-Cues and Atmospheric Scattering Model. Opto-Electron. Eng..

[5] He K., Zhang X., Ren S., Sun J. Deep residual learning for image recognition. Proceedings of the IEEE Conference on Computer Vision and Pattern Recognition.

[6] Dosovitskiy A., Beyer L., Kolesnikov A., Weissenborn D., Zhai X., Unterthiner T., Dehghani M., Minderer M., Heigold G., Gelly S. (2020). An image is worth 16×16 words: Transformers for image recognition at scale. arXiv.

[7] Cui Y., Wang Q., Li C., Ren W., Knoll A. (2025). EENet: An effective and efficient network for single image dehazing. Pattern Recognit..

[8] Qin X., Wang Z., Bai Y., Xie X., Jia H. FFA-Net: Feature Fusion Attention Network for Single Image Dehazing. Proceedings of the AAAI Conference on Artificial Intelligence (AAAI).

[9] Wang Y., Yan X., Wang F.L., Xie H., Yang W., Zhang X.P., Qin J., Wei M. (2024). UCL-Dehaze: Toward real-world image dehazing via unsupervised contrastive learning. IEEE Trans. Image Process..

[10] Kirillov A., Mintun E., Ravi N., Mao H., Rolland C., Gustafson L., Xiao T., Whitehead S., Berg A.C., Lo W.Y. Segment Anything. Proceedings of the IEEE/CVF International Conference on Computer Vision.

[11] Ravi N., Gabeur V., Hu Y.T., Hu R., Ryali C., Ma T., Khedr H., Rädle R., Rolland C., Gustafson L. (2024). SAM 2: Segment anything in images and videos. arXiv.

[12] He K., Sun J., Tang X. (2010). Single image haze removal using dark channel prior. IEEE Trans. Pattern Anal. Mach. Intell..

[13] Zhu Q., Mai J., Shao L. (2015). A fast single image haze removal algorithm using color attenuation prior. IEEE Trans. Image Process..

[14] Berman D., Avidan S. Non-local image dehazing. Proceedings of the IEEE Conference on Computer Vision and Pattern Recognition.

[15] Shi F., Jia Z., Zhou Y. (2025). Zero-Shot Sand–Dust Image Restoration. Sensors.

[16] Ren W., Pan J., Zhang H., Cao X., Yang M.H. (2020). Single image dehazing via multi-scale convolutional neural networks with holistic edges. Int. J. Comput. Vis..

[17] Li B., Peng X., Wang Z., Xu J., Feng D. AOD-Net: All-in-one dehazing network. Proceedings of the IEEE International Conference on Computer Vision.

[18] Zhang H., Patel V.M. Densely connected pyramid dehazing network. Proceedings of the IEEE Conference on Computer Vision and Pattern Recognition.

[19] Li T., Liu Y., Ren W., Shiri B., Lin W. (2025). Single Image Dehazing Using Fuzzy Region Segmentation and Haze Density Decomposition. IEEE Trans. Circuits Syst. Video Technol..

[20] Liu X., Ma Y., Shi Z., Chen J. GridDehazeNet: Attention-based multi-scale network for image dehazing. Proceedings of the IEEE/CVF International Conference on Computer Vision.

[21] Wu H., Qu Y., Lin S., Zhou J., Qiao R., Zhang Z., Xie Y., Ma L. Contrastive learning for compact single image dehazing. Proceedings of the IEEE/CVF Conference on Computer Vision and Pattern Recognition.

[22] Hong M., Liu J., Li C., Qu Y. (2022). Uncertainty-driven dehazing network. Proc. Aaai Conf. Artif. Intell..

[23] Chen Z., He Z., Lu Z.M. (2024). DEA-Net: Single image dehazing based on detail-enhanced convolution and content-guided attention. IEEE Trans. Image Process..

[24] Wang X., Yang G., Ye T., Liu Y. (2025). Dehaze-RetinexGAN: Real-World Image Dehazing via Retinex-based Generative Adversarial Network. Proc. Aaai Conf. Artif. Intell..

[25] Son D.M., Huang J.R., Lee S.H. (2025). Image Sand–Dust Removal Using Reinforced Multiscale Image Pair Training. Sensors.

[26] Zhang S., Ren W., Tan X., Wang Z.-J., Liu Y., Zhang J., Zhang X., Cao X. (2021). Semantic-aware dehazing network with adaptive feature fusion. IEEE Trans. Cybern..

[27] Cheng Z., You S., Ila V., Li H. (2018). Semantic single-image dehazing. arXiv.

[28] Song Y., Yang C., Shen Y., Wang P., Huang Q., Kuo C.C.J. (2018). SPG-Net: Segmentation prediction and guidance network for image inpainting. arXiv.

[29] Zhang Q., Liu X., Li W., Chen H., Liu J., Hu J., Xiong Z., Yuan C., Wang Y. Distilling semantic priors from SAM to efficient image restoration models. Proceedings of the IEEE/CVF Conference on Computer Vision and Pattern Recognition.

[30] Li S., Liu M., Zhang Y., Chen S., Li H., Dou Z., Chen H. SAM-Deblur: Let Segment Anything boost image deblurring. Proceedings of the ICASSP 2024–IEEE International Conference on Acoustics, Speech and Signal Processing.

[31] Liu H., Shao M., Wan Y., Liu Y., Shang K. (2025). SeBIR: Semantic-guided burst image restoration. Neural Netw..

[32] Li J., Wen Y., He L. ScConv: Spatial and channel reconstruction convolution for feature redundancy. Proceedings of the IEEE/CVF Conference on Computer Vision and Pattern Recognition.

[33] Wang Y., Xiong J., Yan X., Wei M. (2023). USCFormer: Unified transformer with semantically contrastive learning for image dehazing. IEEE Trans. Intell. Transp. Syst..

[34] Zhao H., Gallo O., Frosio I., Kautz J. (2016). Loss functions for image restoration with neural networks. IEEE Trans. Comput. Imaging.

[35] Li B., Ren W., Fu D., Tao D., Feng D., Zeng W., Wang Z. (2018). Benchmarking single-image dehazing and beyond. IEEE Trans. Image Process..

[36] Ancuti C.O., Ancuti C., Sbert M., Timofte R. DENSE-HAZE: A benchmark for image dehazing with dense-haze and haze-free images. Proceedings of the 2019 IEEE International Conference on Image Processing (ICIP).

[37] Ancuti C.O., Ancuti C., Timofte R. NH-HAZE: An image dehazing benchmark with non-homogeneous hazy and haze-free images. Proceedings of the IEEE/CVF Conference on Computer Vision and Pattern Recognition Workshops.

[38] Li L., Song S., Lv M., Jia Z., Ma H. (2025). Multi-Focus Image Fusion Based on Fractal Dimension and Parameter Adaptive Unit-Linking Dual-Channel PCNN in Curvelet Transform Domain. Fractal Fract..

[39] Lv M., Song S., Jia Z., Li L., Ma H. (2025). Multi-Focus Image Fusion Based on Dual-Channel Rybak Neural Network and Consistency Verification in NSCT Domain. Fractal Fract..

[40] Cao Z.H., Liang Y.J., Deng L.J., Vivone G. (2025). An Efficient Image Fusion Network Exploiting Unifying Language and Mask Guidance. IEEE Trans. Pattern Anal. Mach. Intell..

[41] Wang Z., Bovik A.C., Sheikh H.R., Simoncelli E.P. (2004). Image quality assessment: From error visibility to structural similarity. IEEE Trans. Image Process..

[42] Sharma G., Wu W., Dalal E.N. (2005). The CIEDE2000 color-difference formula: Implementation notes, supplementary test data, and mathematical observations. Color Res. Appl..

[43] Choi L.K., You J., Bovik A.C. (2015). Referenceless prediction of perceptual fog density and perceptual image defogging. IEEE Trans. Image Process..

[44] Mittal A., Soundararajan R., Bovik A.C. (2012). Making a “completely blind” image quality analyzer. IEEE Signal Process. Lett..

[45] Venkatanath N., Praneeth D., Sumohana S.C., Swarup S.M. Blind image quality evaluation using perception based features. Proceedings of the 2015 Twenty First National Conference on Communications (NCC).

[46] Mittal A., Moorthy A.K., Bovik A.C. (2012). No-reference image quality assessment in the spatial domain. IEEE Trans. Image Process..

[47] Guo C.L., Yan Q., Anwar S., Cong R., Ren W., Li C. Image dehazing transformer with transmission-aware 3D position embedding. Proceedings of the IEEE/CVF Conference on Computer Vision and Pattern Recognition.

[48] Shen H., Zhao Z.Q., Zhang Y., Zhang Z. Mutual information-driven triple interaction network for efficient image dehazing. Proceedings of the 31st ACM International Conference on Multimedia.

[49] Wu R.Q., Duan Z.P., Guo C.L., Chai Z., Li C. RIDCP: Revitalizing real image dehazing via high-quality codebook priors. Proceedings of the IEEE/CVF Conference on Computer Vision and Pattern Recognition.

[50] Zheng Y., Zhan J., He S., Dong J., Du Y. Curricular contrastive regularization for physics-aware single image dehazing. Proceedings of the IEEE/CVF Conference on Computer Vision and Pattern Recognition.

[51] Lu L., Xiong Q., Xu B., Chu D. MixDehazeNet: Mix structure block for image dehazing network. Proceedings of the 2024 International Joint Conference on Neural Networks (IJCNN).

[52] Fu J., Liu S., Liu Z., Guo C.L., Park H., Wu R., Wang G., Li C. Iterative Predictor-Critic Code Decoding for Real-World Image Dehazing. Proceedings of the IEEE/CVF Conference on Computer Vision and Pattern Recognition.

